# Nasal Retinal Degeneration Is a Feature of a Subset of *CRX*-Associated Retinopathies

**DOI:** 10.3390/genes17010050

**Published:** 2026-01-01

**Authors:** Michael T. Massengill, Tamara Juvier Riesgo, Janet L. Davis, Carlos E. Mendoza-Santiesteban, Brian E. Goldhagen, Byron L. Lam, Ninel Z. Gregori

**Affiliations:** 1 Bascom Palmer Eye Institute, Miller School of Medicine, University of Miami, Miami, FL 33136, USA; txj362@med.miami.edu (T.J.R.); jdavis@med.miami.edu (J.L.D.); cmendoza@miami.edu (C.E.M.-S.); bxg150@miami.edu (B.E.G.); blam@med.miami.edu (B.L.L.); ngregori@med.miami.edu (N.Z.G.); 2 Department of Ophthalmology, Veteran’s Affairs Hospital, Miami, FL 33125, USA

**Keywords:** inherited retinal degeneration, *cone-rod homeobox* gene, *CRX*, maculopathy, cone–rod dystrophy, Leber congenital amaurosis

## Abstract

**Background/Objectives**: Genetic variants in the cone–rod homeobox (*CRX*) gene, a transcription factor critical for the differentiation, function, and survival of photoreceptors, are a rare cause of inherited retinal diseases (IRDs). *CRX*-associated retinopathies can produce variable phenotypes, including Leber congenital amaurosis (LCA), maculopathy (M), cone-rod dystrophy (CRD), and rod-cone dystrophy (RCD), such as retinitis pigmentosa (RP). Based on clinical observations at our eye institute, we hypothesized that nasal retinal degeneration is a feature of *CRX*-associated maculopathy and M/CRD. **Methods**: We performed an IRB-approved, retrospective review of patients at our eye institute with *CRX*-associated retinopathy to assess the frequency of nasal degeneration and potential genotype–phenotype correlations. **Results**: A total of 15 patients with a *CRX*-associated retinopathy and meeting the inclusion criteria were identified (LCA 3, RCD/RP 2, M/CRD 10). Overall, nasal degeneration occurred in 8 of 15 patients (53.3%) in the cohort. Nasal retinal degeneration was seen in the M/CRD (6 of 10; 60.0%) as well as LCA groups (2 of 3; 66.6%). No significant differences in age, gender, or presenting visual acuity were observed between patients with and without nasal degeneration. Genetic variants associated with nasal degeneration are localized to both the homeobox motif and activation domain. Intronic variants were relatively more common in patients with nasal degeneration, while missense variants predominated in those without, although these differences were not statistically significant. **Conclusions**: We conclude that nasal degeneration is a feature of a subset of *CRX*-associated phenotypes, affects both genders, and can be caused by genetic variants in multiple locations and of various subtypes.

## 1. Introduction

Inherited retinal diseases (IRDs) are a group of genetically heterogenous blinding disorders that affect the function and survival of the cells of the retina and choroid. Depending on the distribution and type of cells affected, patients may experience symptoms of decreased visual acuity, central scotoma, dyschromatopsia, photophobia, and hemeralopia with cone photoreceptor dysfunction, or nyctalopia, impaired dark adaptation, and peripheral visual field loss with rod photoreceptor dysfunction. Over 300 genes, when affected by pathogenic variants, can lead to an IRD phenotype (https://retnet.org, accessed on 1 November 2025).

A rare subtype of IRDs is caused by pathogenic variants in the cone–rod homeobox-containing gene (*CRX*; OMIM #602225), which has been mapped to the 19q13.33 chromosome and was first implicated in IRDs by Evans et al. in 1994 [1]. The *CRX* gene is comprised of four exons, the first of which is non-coding, and encodes a 299 amino acid transcription factor that is expressed in photoreceptors, retinal pigment epithelium (RPE), and the pineal gland [2]. The protein plays a central role in the differentiation and maintenance of the function of photoreceptor cells [3]. The transcription factor is composed of an N-terminal DNA-binding homeobox domain (residues 39–99), a WSP motif (residues 158–170) within a transactivating domain towards its C-terminus (residues 113–284), and an orthodenticle-related homeobox (OTX) tail at the C-terminus (residues 284–295) [4,5]. The majority of pathogenic variants cluster within the homeobox and transactivation domains, which exhibit minimal tolerance for genetic variation [6].

As with IRDs as a group, *CRX*-associated retinopathies are genetically heterogenous with over 130 causative variants identified [7] (http://www.hgmd.cf.ac.uk, accessed on 1 November 2025). Most *CRX*-associated retinopathies are presumed to be inherited in an autosomal dominant fashion due to gain-of-function and dominant negative effects of the mutated protein [4,8]. Rare cases of haploinsufficiency have, however, been reported [9]. The diversity of *CRX*-associated phenotypes mirrors the underlying genetic heterogeneity and manifests as macular dystrophy (M), cone or cone–rod dystrophies (CRD), rod-cone dystrophy (RCD) or retinitis pigmentosa (RP), and Leber congenital amaurosis (LCA) [2]. Given the tremendous genotypic and phenotypic variability, continued description of *CRX*-associated retinopathies is required for complete characterization of the range of possible genotype–phenotype combinations. To this end, a distinct phenotype of cone and/or rod dysfunction as well as bifocal degeneration involving the macula and a separate area of nasal retina was recently reported to be associated with genetic variants in *CRX* by Lin et al. [10]. Given the scarcity of published literature on *CRX*-associated phenotypes, we conducted a case series review of patients with *CRX*-associated retinopathy at the Bascom Palmer Eye Institute to assess the frequency of nasal retinal degeneration and potential genotype–phenotype correlations.

## 2. Materials and Methods

We conducted an Institutional Review Board (IRB) approved, retrospective chart review of patients evaluated in the Inherited Retinal Disease (IRD) service at the Bascom Palmer Eye Institute. We reviewed patients who exhibited an IRD phenotype and had either a positive or a variant of uncertain significance (VUS) result in the *CRX* gene that were consistent with their clinical presentation.

Patients received an IRD diagnosis based on multimodal testing, which included any of the following: color fundus photos, spectral-domain optical coherence tomography (SD-OCT), fundus autofluorescence (FAF), Humphrey or Goldmann visual field testing, full-field electroretinography (ffERG), and/or multifocal ERG (mfERG). Panel-based genetic testing was conducted through commercial laboratories (Invitae/Spark Therapeutics, Molecular Vision Laboratory, Casey Eye Institute, Blueprint Genetics) after obtaining informed consent. Splicing analysis was performed with Genomnins, Human Splicing Finder Pro Systems [11].

The presence of nasal degeneration was qualitatively assessed by examining color fundus photographs and FAF images for pigmentary changes, atrophy, or abnormal autofluorescence signals in the nasal retina. Patients were excluded if fundus photography, SD-OCT images, and/or FAF images of the nasal retina were not available.

Demographic (age, sex, race/ethnicity), clinical (best-corrected visual acuity [BCVA]), and genotype data were collected for analysis. Statistical analyses were performed using: (1) Student’s *t*-test (unpaired, 2-tailed, assumed equal variance) to assess for differences in presenting age and BCVA between groups (Microsoft Excel, Microsoft, Redmond, WA, USA), and (2) Chi-square analysis to assess for differences in the frequency distributions of sex, genotypes, and phenotypes between groups (GraphPad, San Diego, CA, USA). A *p*-value of <0.05 was considered to be statistically significant.

Figures and tables were assembled using Microsoft Excel and PowerPoint.

## 3. Results

**Figure 1:**
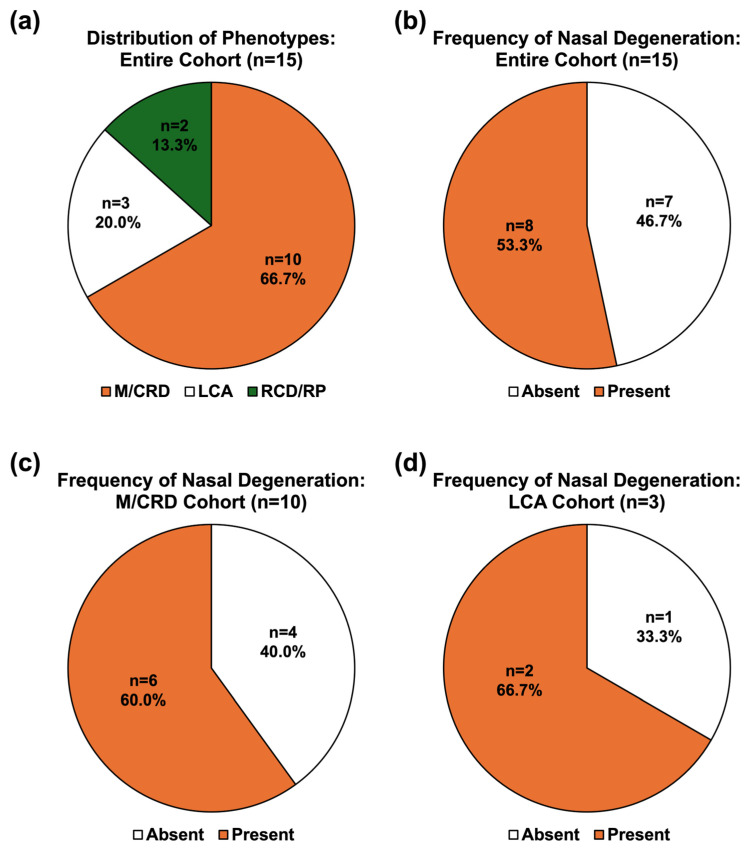
**Distribution of Clinical Phenotypes**. (**a**) Among 15 patients with *CRX*-associated retinopathy that met the inclusion criteria, 66.7% (n = 10) exhibited a maculopathy/cone-rod dystrophy (M/CRD) phenotype, 20.0% (n = 3) had a Leber Congenital Amaurosis (LCA) phenotype, and 13.3% (n = 2) presented with a rod-cone dystrophy/retinitis pigmentosa (RCD/RP) phenotype. (**b**) Nasal degeneration was present in 8 of the 15 patients (53.3%) in the entire cohort. (**c**) Of those with the M/CRD phenotype, 60.0% (n = 6) demonstrated nasal retinal degeneration, while 40.0% (n = 4) did not. (**d**) Within the cohort of LCA patients (n = 3), 2 patients exhibited nasal degeneration (66.7%). No patients belonging to our limited RCD/RP phenotype cohort had nasal degeneration.

**Figure 2:**
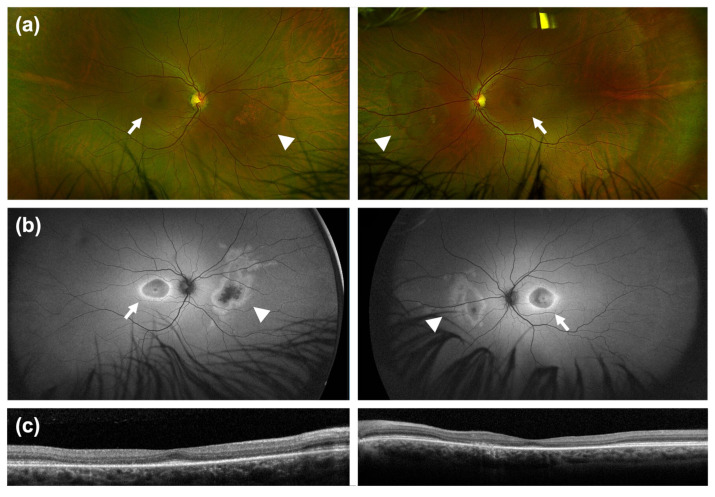
**Representative multimodal imaging of Patient 4—*CRX* associated M/CRD due to a p.Ser150Ter (c.449C>G) pathogenic variant.** 32 year-old male patient with a BCVA of 20/30 OD and 20/40 OS. Genetic testing was performed with Invitae (Inherited Retinal Disorders Panel). Ultrawide-field fundus photography (**a**) and fundus autofluorescence (**b**) demonstrate macular atrophy in a bull’s eye configuration (white arrows) along with a separate area of nasal retinal degeneration (white arrowheads). (**c**) SD-OCT reveals outer retinal atrophy corresponding to the region of bull’s eye maculopathy.

A total of 21 patients either had a pathogenic/likely pathogenic variant or a VUS genetic test result for the *CRX* gene. One patient was excluded after the variant was re-classified as likely benign. A second patient was excluded because their pedigree demonstrated a likely X-linked inheritance pattern, which is inconsistent with *CRX*-associated retinopathies. Four patients did not have FAF imaging capturing the nasal retina and were thus excluded. A total of 15 patients ultimately met the inclusion criteria. Among this cohort (n = 15), 66.7% (n = 10) had a maculopathy/cone–rod dystrophy (M/CRD) phenotype, 20.0% (n = 3) had an LCA phenotype, and 13.3% (n = 2) had a rod-cone dystrophy/retinitis pigmentosa (RCD/RP) phenotype (Figure 1a). Nasal degeneration was present in 8 of 15 patients (53.3%) in the entire *CRX*-associated retinopathy cohort (Figure 1b). Six of the 10 patients (60.0%) with the M/CRD phenotype (Figure 1c) and 2 of the 3 patients (66.6%) with the LCA phenotypes (Figure 1d) exhibited nasal retinal degeneration, respectively. Of note, no patients in our limited RCD/RP cohort had nasal degeneration (n = 2). Multimodal imaging of the patients with the M/CRD or LCA phenotype with nasal degeneration is available in Figure 2 as well as Supplementary Figures S1–S7.

Demographic and genetic data for individuals with the RCD/M or LCA phenotype and nasal degeneration are shown in Table 1. Of note, patients 5 and 6 are first-degree relatives (daughter/mother) and thus have the same *CRX* VUS (c.100 + 3G>C). Since both family members exhibited a similar phenotype consistent with M/CRD, we considered their VUS as disease-causing. Furthermore, bioinformatic analysis predicted that the c.100 + 3G>C variant produced an alteration to the wild-type splice donor site, most probably affecting splicing (Genomnins, Human Splicing Finder Pro Systems) [11].

**Figure 3:**
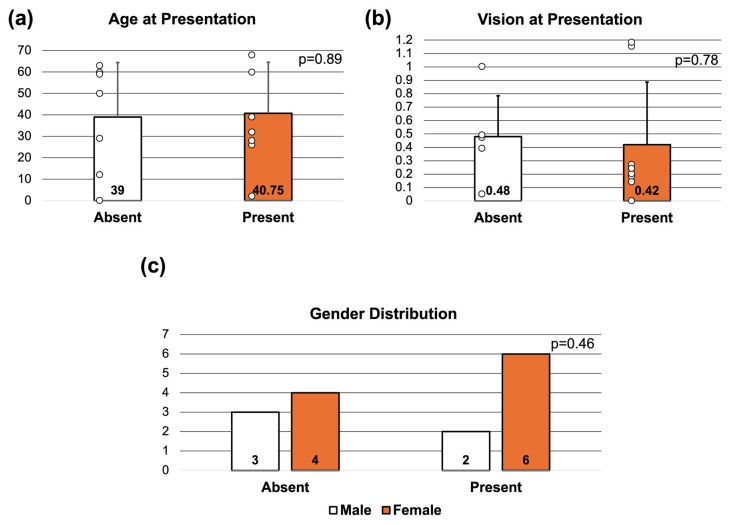
**Comparison of Patient Demographic Data Based on the Presence or Absence of Nasal Degeneration**. (**a**) The average age pf patients without (n = 7) and (n = 8) nasal degeneration was 39.0 and 40.75 years, respectively, with no statistically significant difference. (**b**) The average LogMAR visual acuity of patients without (n = 6) and with (n = 8) nasal degeneration was 0.48 and 0.42, respectively, also with no statistically significant difference. Of note, one patient in the absent group was too young for visual acuity measurement, thus was excluded from this analysis. Student’s *t*-test was used to compare the average age and LogMAR visual acuity between groups. (**c**) A higher proportion of female patients was observed in the nasal degeneration group (6 of patients when present (75%) vs. 4 of 7 patients when absent (57%)), however there was no statistically significant difference between gender distribution of each group. Chi-squared analysis was used to compare gender distribution between groups.

The average age at presentation for patients with (40.75 ± 23.87 years) versus without (39.00 ± 25.46 years) nasal degeneration was not statistically different between groups (*p* = 0.89) (Figure 3a). The average vision at presentation of patients with (0.42 ± 0.47 logMAR) versus without (0.48 ± 0.30 logMAR) nasal was also not statistically different (*p* = 0.78) (Figure 3b). In the entire cohort, female gender was more frequently observed in patients with nasal degeneration (6 of 8 patients, 75%) versus those without (4 of 7 patients, 57%). A similar gender distribution was seen in the M/CRD cohort. The gender distributions, however, were not statistically different when comparing patients with and without nasal degeneration (*p* = 0.46) (Figure 3c).

**Figure 4:**
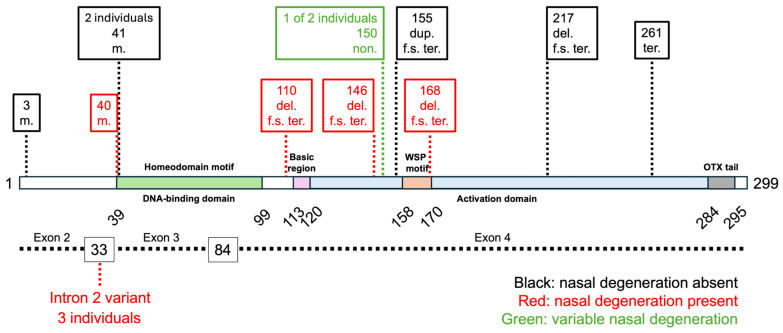
**Locations of Variants Associated with Nasal Degeneration Across the Protein and Exon Sequences of *CRX***. The amino acid sequence is represented by the horizontal bar at the top, and the exon sequence is shown as a black dashed line across the bottom. Variants associated with the presence of nasal degeneration are shown in red (n = 7), while those associated with its absence are shown in black (n = 6). Variant c.449C > G (p.Ser150Ter), shown in green, presented as M/CRD with nasal degeneration in one patient and as RCD/RP without nasal degeneration in a different patient in the cohort. Notably, two individuals had variants at the amino acid position 41 and three individuals had variants in intron 2. A list of variants can be found in Supplementary Table S1. Abbreviations: m. = missense; non. = nonsense; del. = deletion; dup. = duplication; f.s. = frameshift; ter. = premature stop codon.

Genetic variants associated with nasal degeneration were located within the homeodomain motif, intron 2, and the N-terminal region of the transactivation domain in our cohort (Figure 4). When including variants that produced a similar phenotype from Lin et al. (n = 6) [10], the variants appeared to be distributed widely along the conserved homeodomain and activation regions of the protein and gene sequences (Supplementary Figure S8). Variant sequences are available in Supplementary Table S1. Interestingly, the nonsense variant p.Ser150Ter presented as M/CRD with nasal degeneration in one patient and as RCD/RP without nasal degeneration in a different patient in our cohort (Figure 2, Supplementary Figure S9). We also observed that the patient with the missense variant p.Arg41Trp in our cohort did not exhibit nasal degeneration (Supplementary Figure S10); however, this same variant did cause nasal degeneration in the Lin et al. report [10].

**Figure 5:**
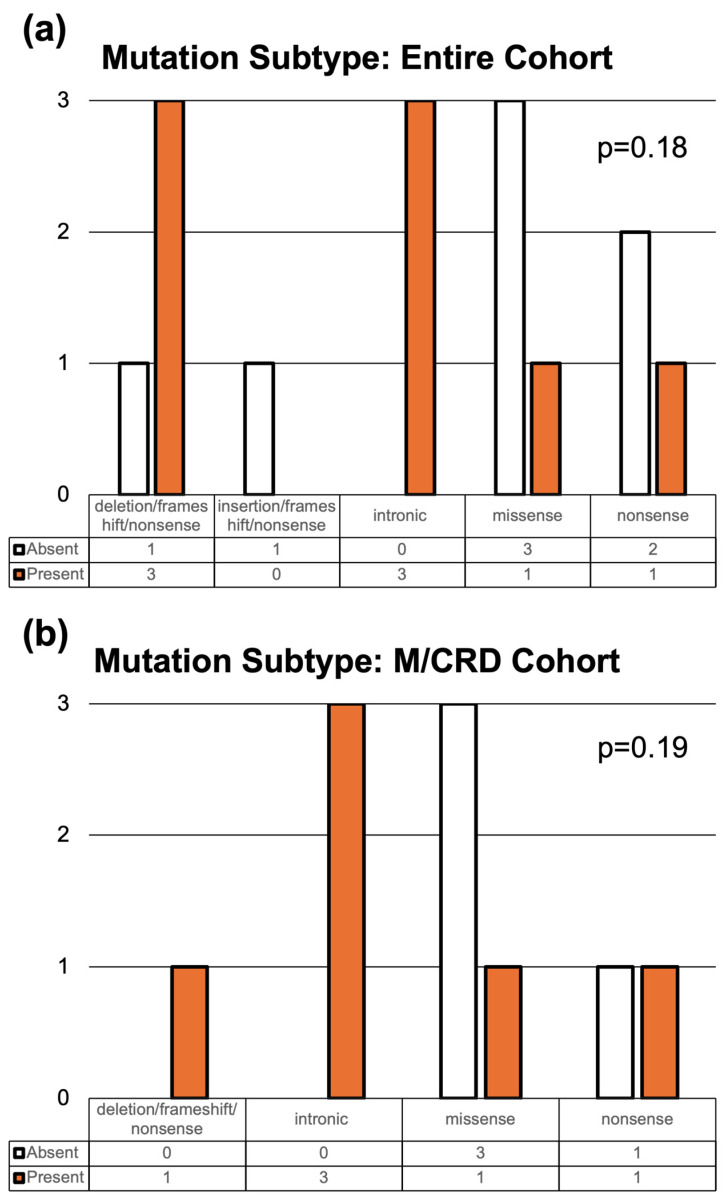
***CRX* Variant Subtype Stratified by Presence or Absence of Nasal Degeneration.** (**a**) Bar graph showing the frequency of variant subtypes in the entire cohort, stratified by the presence (n = 8; orange bar) or absence (n = 7; white bar) of nasal degeneration. (**b**) Bar graph showing the frequency of variant subtypes in M/CRD cohort, stratified by the presence (n = 6; orange bar) or absence (n = 4) of nasal degeneration. Although intronic and missense appeared more frequently when nasal degeneration was present or absent, respectively, the distributions of variant subtypes were not statistically different between groups. Statistical analysis was performed using Chi-squared testing.

We next compared the distributions of variant subtype (e.g., missense, nonsense, intronic) when nasal degeneration was present versus absent to determine if a particular subtype was correlated with a specific phenotype. When analyzing the entire cohort (Figure 5a) as well as the M/CRD cohort alone (Figure 5b), we found that the distributions of variant subtypes when nasal degeneration was present versus absent were not statistically different. Nonetheless, intronic variants were only encountered when nasal degeneration was present (although two of the three patients were first-degree relatives—patients 5 and 6), and missense variants were more common when nasal degeneration was absent.

## 4. Discussion

*CRX* genetic variants were first associated with the CRD phenotype; however, typical phenotypes are now accepted to include maculopathy (M), CRD, RCD, or RP, and LCA [2]. Individual case reports have described other unique *CRX*-associated retinopathy phenotypes, including occult macular dystrophy [12], as well as phenotypes resembling benign concentric annular macular dystrophy [13], pigmented paravenous retinochoroidal atrophy [14], X-linked retinoschisis [15], benign concentric annular macular dystrophy [16], and macular coloboma-like lesions [17,18]. Our findings, which support those of Lin et al. [10], suggest that nasal retinal degeneration is a feature observed in a subset of M/CRD caused by *CRX* variants, which occurred at a rate of 60.0% in our M/CRD cohort. In their study, Lin et al. found a discrete area of nasal retinal degeneration in 6 of 50 patients (12%) with variants in *CRX*, all of which exhibited central retinal degeneration and a cone or cone–rod pattern of dysfunction on electroretinography [10]. The prognostic value of nasal degeneration remains unclear, as neither age nor visual acuity at presentation differed significantly between patients with and without this finding. Additionally, we noted that 2 of the 3 cases with LCA also had a distinct area of nasal degeneration, suggesting that LCA patients may exhibit this finding as well. We did not observe nasal degeneration in our RCD/RP cohort; nonetheless, our cohort consistent of only two patients. All in all, we identified 8 cases of nasal retinal degeneration in our cohort of 15 patients (53.3%) with *CRX*-associated retinopathy.

Importantly, the cohort of *CRX*-mutated patients with nasal degeneration reported by Lin et al. described only male patients (n = 6). As a result, they posited that there may be sex-related differences in the nasal degeneration phenotype [10]. In contrast, our cohort with nasal degeneration predominantly consisted of female patients (6 of 8 patients with nasal degeneration). Our findings indicate that the nasal retinal degeneration phenotype can occur in both male and female patients. Consequently, the low prevalence of *CRX*-associated retinopathies likely contributed to the skewed gender distributions in both our study and the report by Lin et al. [10]. To this end, larger cohorts will be necessary to clarify any true sex predilection.

Genotype–phenotype correlations in IRDs are known to involve a complex interplay of factors, including the primary pathogenic variant, modifier genes, epigenetic influences, environmental exposures, and stage of disease [19]. In our case series, patients 5 and 6 are first-degree relatives (daughter/mother) who both exhibited nasal retinal degeneration (Supplementary Figures S4 and S5), thus suggesting genetic or environmental factors may be contributory. These patients also illustrate that nasal degeneration may become more pronounced with disease progression, as the mother (patient 6) exhibited more noticeable nasal degeneration than the daughter (patient 5). Importantly, we observed that two *CRX* variants (p.Ser150Ter and p.Arg41Trp) were variably associated with nasal degeneration when compared either to another patient within our cohort or to one in the cohort described by Lin et al. [10]. This suggests that the variant alone may not be sufficient to produce this phenotype. Indeed, the same variant can produce varying phenotypes across and within families. For example, the c.425A>G can cause early-onset retinal dystrophy in some individuals [20] or late-onset RP in others [21]. Additional sources of phenotypic variability include disease progression with age [22], variable expressivity [23], somatic mosaicism [24], incomplete penetrance [25], and ethnic disparities [26].

The reason for the underlying susceptibility of the nasal retina to pathologic variants of *CRX* remains unknown. This pattern may manifest because of environmental light exposure, which has been observed in animal models of RP [27] and in humans with pathogenic variants in rhodopsin [28]. Alternatively, because the *CRX* protein is expressed in both cone and rod photoreceptors, degeneration would be expected to occur in areas with the highest densities of these cell types in the retina. Degeneration would therefore be anticipated to be most severe in the macula and approximately 20 degrees eccentric to the fovea—and particularly nasally—for cones and rods, respectively [29]. Finally, spatial differences in *CRX* expression may contribute to accelerated retinal degeneration in areas with higher expression. Nonetheless, to our knowledge, this hypothesis has not yet been explored.

Literature evidence suggests that the location and subtype of a *CRX* variant may correlate with the resulting phenotype. Missense variants occur most frequently within the homeobox domain, whereas frameshift variants and those creating premature stop codons are more often found in the C-terminus [6,7]. Furthermore, variants within the homeodomain are more frequently associated with M or CRD, whereas those closer to the C-terminus display a more diverse phenotypic spectrum [7]. In our cohort, *CRX* variants associated with the nasal degeneration phenotype were localized to the homeodomain and the N-terminal portion of the activation domain; however, when including *CRX* variants causing the nasal degeneration phenotype from the recent study by Lin et al. [10], the variants appear to be distributed across the majority of the protein sequence, including both the homeobox domain and the N- and C-termini of the activation domain (Supplementary Figure S8). This suggests that no specific locus currently predicts nasal degeneration. We did not find a correlation between a particular variant subtype (e.g., missense, nonsense) and the presence or absence of nasal degeneration. Nonetheless, intronic variants were only encountered when nasal degeneration was present, and missense variants were more commonly encountered in our cohort when nasal degeneration was absent.

Although our study reports novel findings, there are several limitations. We utilized multiple types of cameras to capture fundus photography and FAF images, rather than a standardized imaging system. This was due to the inclusion of patients imaged by different providers. Additionally, given the small sample size, the recorded prevalence of nasal degeneration modulates across studies, and thus, the true prevalence may not be known as of yet. Indeed, we report an incidence of 53.3%, whereas Lin et al. reported 12% [10]. Furthermore, our analysis is cross-sectional, capturing a single point in time and thus does not provide information regarding disease progression or prognosis. In addition, some patients without nasal degeneration may develop it later with longitudinal follow-up. This may be particularly relevant for the two variants that show variable nasal degeneration in our study, and when compared to the study by Lin et al. [10]. Finally, our study was not designed to identify the factors that contribute to the development of nasal degeneration.

## 5. Conclusions

Analysis of our *CRX*-associated retinopathy cohort suggests that nasal retinal degeneration may be a distinguishing feature in a subset of *CRX*-associated retinopathies. Our data indicate that this phenotype occurs in both genders, can occur with both the M/CRD and LCA phenotypes, and can arise from variants at various loci within the gene. Although the presence of nasal degeneration may point to the underlying genetic etiology as caused by a variant in *CRX*, its prognostic significance remains unknown. Future studies should explore (1) the prognostic significance of nasal degeneration, (2) whether environmental factors or modifier genes are necessary for the development of the nasal degeneration phenotype, (3) whether specific loci and variant subtypes in the *CRX* gene are associated with nasal degeneration in a larger cohort, and (4) whether a subset of RCD/RP patients also exhibit nasal retinal degeneration.

## Figures and Tables

**Table 1:** Demographic, clinical, and genotypic characteristics of patients with nasal retinal degeneration.

			BCVA (Snellen, [logMAR])								
Patient	Gender	Age	OD	OS	Race	Ethnicity	Phenotype	Mutation	Mutation Subtype	Inheritance	Variant Classification
1	Female	2	20/200, [1]	20/400, [1.3]	White	Non-Hispanic or Latino	LCA	c.503_504del, p.Glu168Valfs*5	Deletion/frameshift/nonsense	Heterozygous	Likely pathogenic
2	Female	26	20/20, [0]	20/70, [0.54]	White	Non-Hispanic or Latino	M/CRD	c.329delG, p.Gly110AlafsTer77	Deletion/frameshift/nonsense	Heterozygous	Pathogenic
3	Male	28	20/300, [1.2]	20/300, [1.2]	White	Non-Hispanic or Latino	LCA	c.435delT, p.Leu146CysfsTer41	Deletion/frameshift/nonsense	Heterozygous	Likely pathogenic
4	Male	32	20/30, [0.2]	20/40, [0.3]	White	Hispanic or Latino	M/CRD	c.449C>G, p.Serl50Ter	Nonsense	Heterozygous	Pathogenic
5	Female	39	20/20, [0]	20/20, [0]	White	Non-Hispanic or Latino	M/CRD	c.100+3G>C	Intronic	Heterozygous	VUS
6	Female	60	20/25, [0.1]	20/40, [0.3]	White	Non-Hispanic or Latino	M/CRD	c.100+3G>C	Intronic	Heterozygous	VUS
7	Female	68	20/30, [0.2]	20/25, [0.1]	White	Non-Hispanic or Latino	M/CRD	c.100+3_100+5delGAGinsTTA	Intronic	Heterozygous	Likely pathogenic
8	Female	71	20/30, [0.2]	20/30, [0.2]	White	Hispanic or Latino	M/CRD	c.118C>T, p.Arg40Trp	Missense	Heterozygous	Pathogenic

## Data Availability

Data generated or analyzed during this study are not publicly available due to ethical restrictions related to participant privacy.

## Supplementary Materials

The following supporting information can be downloaded at: https://www.mdpi.com/article/10.3390/genes17010050/s1, Figure S1: Multimodal imaging of a Patient 1—*CRX*-associated LCA due to a p.Glu168Valfs*5 (c.503_504del) likely pathogenic variant; Figure S2: Multimodal imaging of a Patient 2—*CRX*-associated M/CRD due to a p.Gly110AlafsTer77 (c.329delG) pathogenic variant; Figure S3: Multimodal imaging of a Patient 3—*CRX*-associated LCA due to a p.Leu146CysfsTer41 (c.435delT) likely pathogenic variant; Figure S4: Multimodal imaging of a Patient 5—*CRX*-associated M/CRD due to a c.100 + 3G>C variant of uncertain significance; Figure S5: Multimodal imaging of a Patient 6—*CRX*-associated M/CRD due to a c.100+3G>C variant of uncertain significance; Figure S6: Multimodal imaging of a Patient 7—*CRX*-associated M/CRD due to a c.100+3_100 + 5delGAGinsTTA likely pathogenic variant; Figure S7: Multimodal imaging of a Patient 8—*CRX*-associated M/CRD due to a p.Arg40Trp (c.118C>T) pathogenic variant; Figure S8: Locations of Variants Associated with Nasal Degeneration Across the Protein and Exon Sequences, Including Variants Reported by Lin et al.; Figure S9: Multimodal imaging of *CRX*-associated RCD/RP without focal nasal degeneration due to a p.Ser150Ter (c.449C>G) pathogenic variant; Figure S10: Multimodal imaging of *CRX*-associated M/CRD without nasal degeneration due to a p.Arg41Trp (c.121 C>T) pathogenic variant; Table S1: Genetic variant, variant subtype, and variant classification for patients with maculopathy or cone–rod dystrophy in our cohort.

## Abbreviations

The following abbreviations are used in this manuscript:

*CRX*	Cone-rod homeobox
IRD	Inherited retinal diseases
LCA	Leber congenital amaurosis
RCD	Rod-cone dystrophy
RP	Retinitis pigmentosa
M	Maculopathy
CRD	Cone-rod dystrophy
RPE	Retinal pigment epithelium
OTX	Orthodenticle-related homeobox
IRB	Institutional Review Board
VUS	Variant of uncertain significance
SD-OCT	Spectral-domain optical coherence tomography
FAF	Fundus autofluorescence
ffERG	Full-field electroretinography
mfERG	Multifocal electroretinography
M/CRD	Maculopathy/cone-rod dystrophy
RCD/RP	Rod-cone dystrophy/retinitis pigmentosa
