# Supplementary material for: Nasal Retinal Degeneration Is a Feature of a Subset of CRX-Associated Retinopathies
Source: Genes (Basel). 2026 Jan 1;17(1):50. doi: 10.3390/genes17010050 (PMC12841240; doi:10.3390/genes17010050)
Supplement: Supplementary file 1 [file genes-17-00050-s001.zip › genes-4000129-supplementary.pdf]

Supplementary Table S1. Genetic variant, variant subtype, and variant classification for patients with maculopathy or cone-rod dystrophy in our cohort. Patients are stratified by absence (black) or presence (red) of nasal degeneration. Also included are patients (blue) with CRX-associated retinopathy and nasal degeneration reported by *Lin et al.* (doi: 10.1038/s41433-024-03522-2) [10].

Mutation	Mutation Subtype	Variant Classification
Whole gene deletion	Whole gene deletion	Pathogenic
c.8C>T, p.Ala3Val	Missense	VUS
c.100+3G>C	Intronic	VUS
c.100+3G>C	Intronic	VUS
c.100+3_100+5delGAGinsTTA	Intronic	Likely pathogenic
c.118C>T, p.Arg40Trp	Missense	Pathogenic
c.122G>A, p.Arg41Gln	Missense	Likely pathogenic
c.121C>T, p.Arg41Trp	Missense	Pathogenic
c.121C>T, p.Arg41Trp	Missense	Pathogenic
c.127C>T, p.Arg43Cys	Missense	Pathogenic
c.329delG, Gly110AlafsTer77	Deletion/frameshift/nonsense	Pathogenic
c.435delT, p.Leu146CysfsTer41	Deletion/frameshift/nonsense	Likely pathogenic
c.449C>G, p.Serl50Ter	Nonsense	Pathogenic
c.449C>G, p.Serl50Ter	Nonsense	Pathogenic
c.463dupA, p.Thr155AsnfsTer19	Insertion/frameshift/nonsense	Pathogenic
c.503_504del, p.Glu168fs	Deletion/frameshift/nonsense	Likely pathogenic
c.568_590del; p.Pro190GlyfsTer38	Deletion/frameshift/nonsense	Likely pathogenic
c.605del; p.Cys220SerfsTer17	Deletion/frameshift/nonsense	Likely pathogenic
c.615del, p.Ser206ProfsTer13	Deletion/frameshift/nonsense	Likely pathogenic
c.650del, p.Gly217fs	Deletion/frameshift/nonsense	Pathogenic
c.783C>G, p.Tyr261Ter	Nonsense	Pathogenic

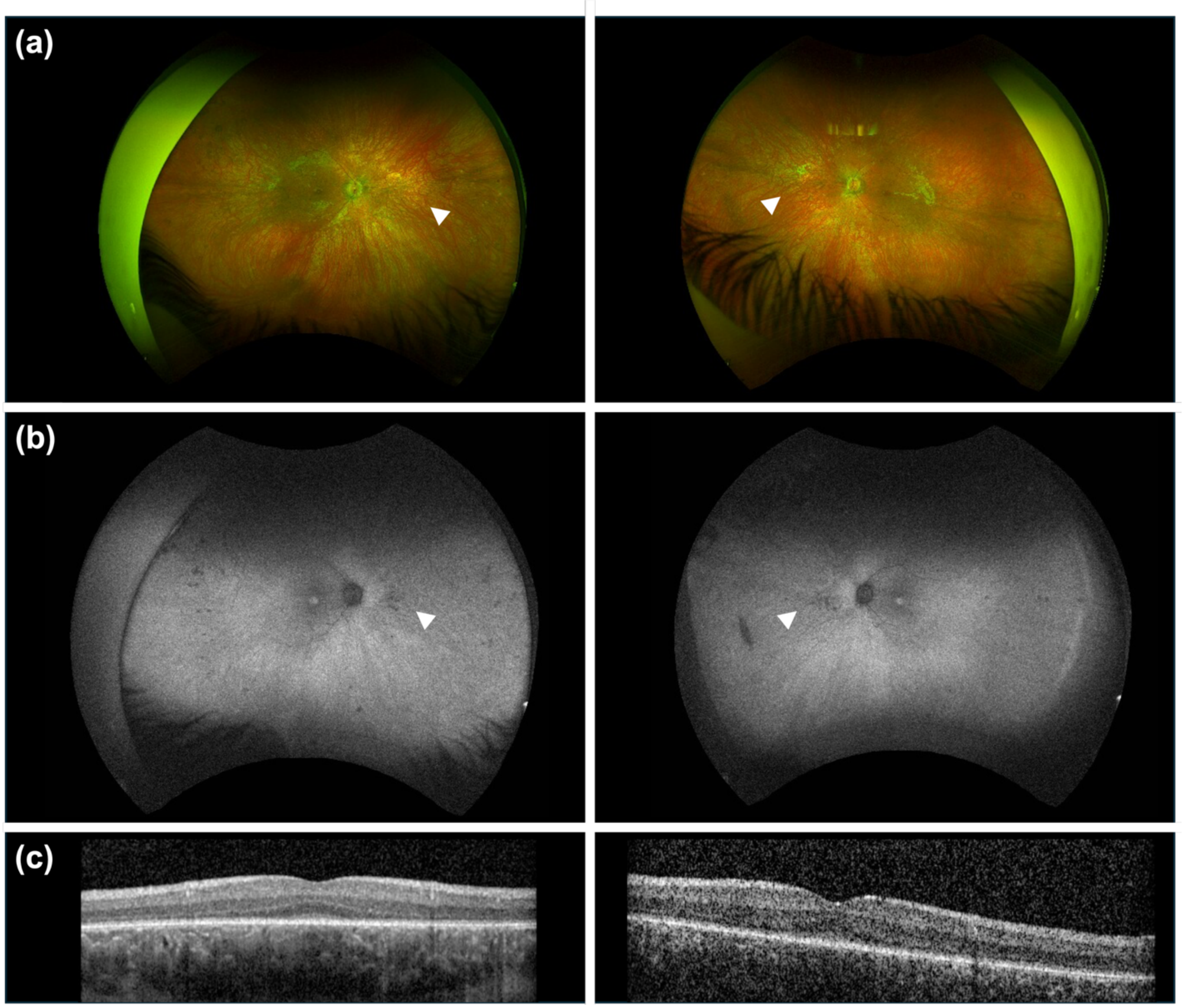

Supplementary Figure S1. Multimodal imaging of a Patient 1 - CRX-associated LCA due to a *p.Glu168Valfs*5 (c.503_504del)* likely pathogenic variant. 2-year-old female patient with a BCVA of 20/200 OD and 20/400 OS. Genetic testing was performed with Prevention Genetics (31 genes tested). Ultrawide-field fundus photography **(a)** and fundus autofluorescence imaging **(b)** show a blonde fundus, diffuse atrophy, and an area of nasal atrophy (white arrowheads). **(c)** Spectral-domain optical coherence tomography (SD-OCT) shows loss of the outer retina, with some retention of these structures in the fovea.

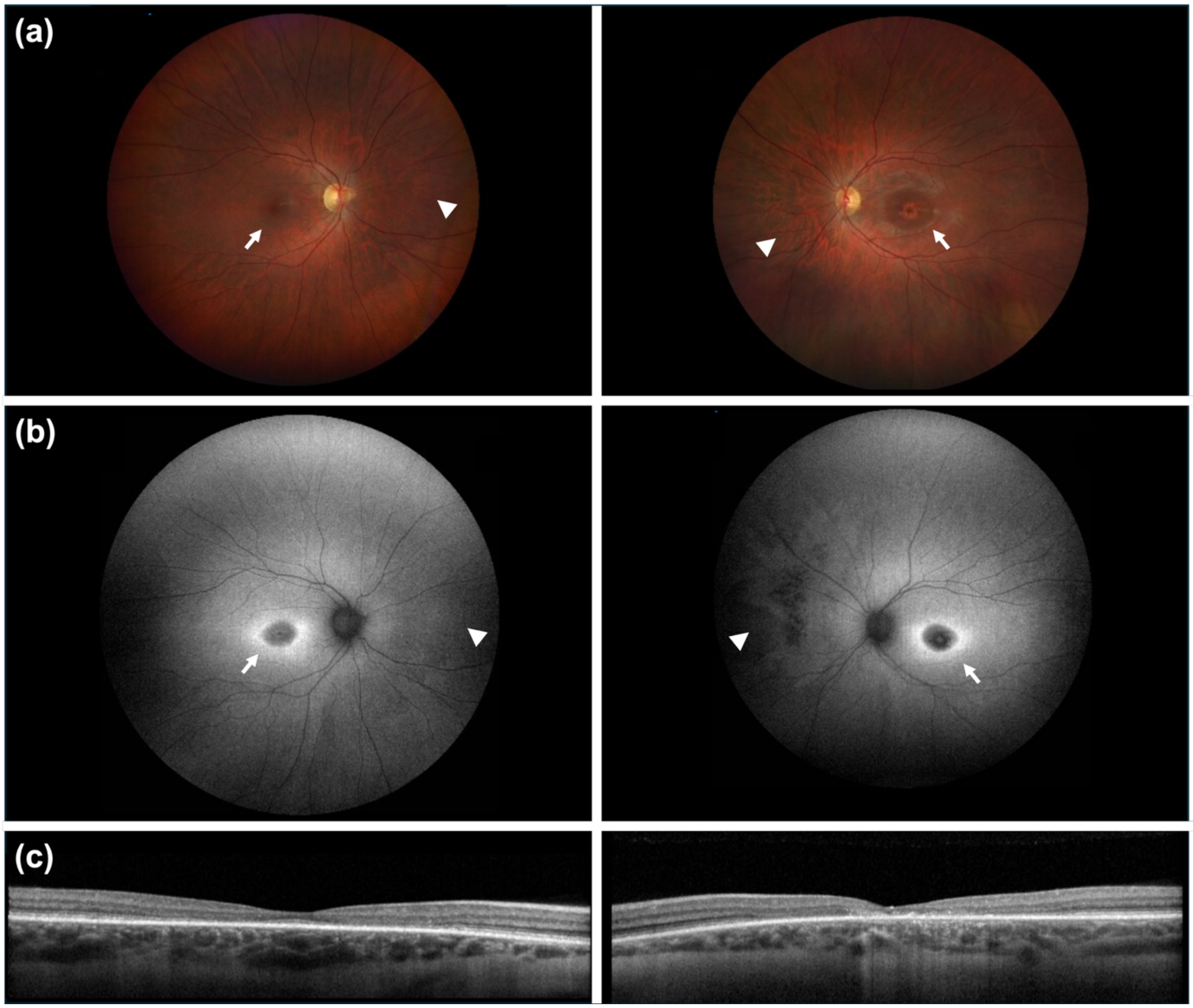

Supplementary Figure S2. Multimodal imaging of a Patient 2 - CRX-associated M/CRD due to a *p.Gly110AlafsTer77* (*c.329delG)* pathogenic variant. 26-year-old female patient with a BCVA of 20/20 OD and 20/70 OS. HVF 30-2 showed central scotoma OU and an area of decreased sensitivity corresponding to the peripapillary nasal retina OS. Full-field ERG demonstrated normal rod responses as well as delayed and reduced cone responses. Genetic testing was performed with Molecular Vision Lab (Vision Panel v7). Ultrawide-field fundus photography **(a)** and fundus autofluorescence **(b)** reveal macular atrophy with a characteristic bull's eye pattern (white arrows), as well as a distinct zone of nasal retinal degeneration (white arrowheads). The nasal involvement is more clearly delineated on fundus autofluorescence in the left eye. **(c)** SD-OCT shows outer retinal thinning corresponding to the area of bull's eye maculopathy.

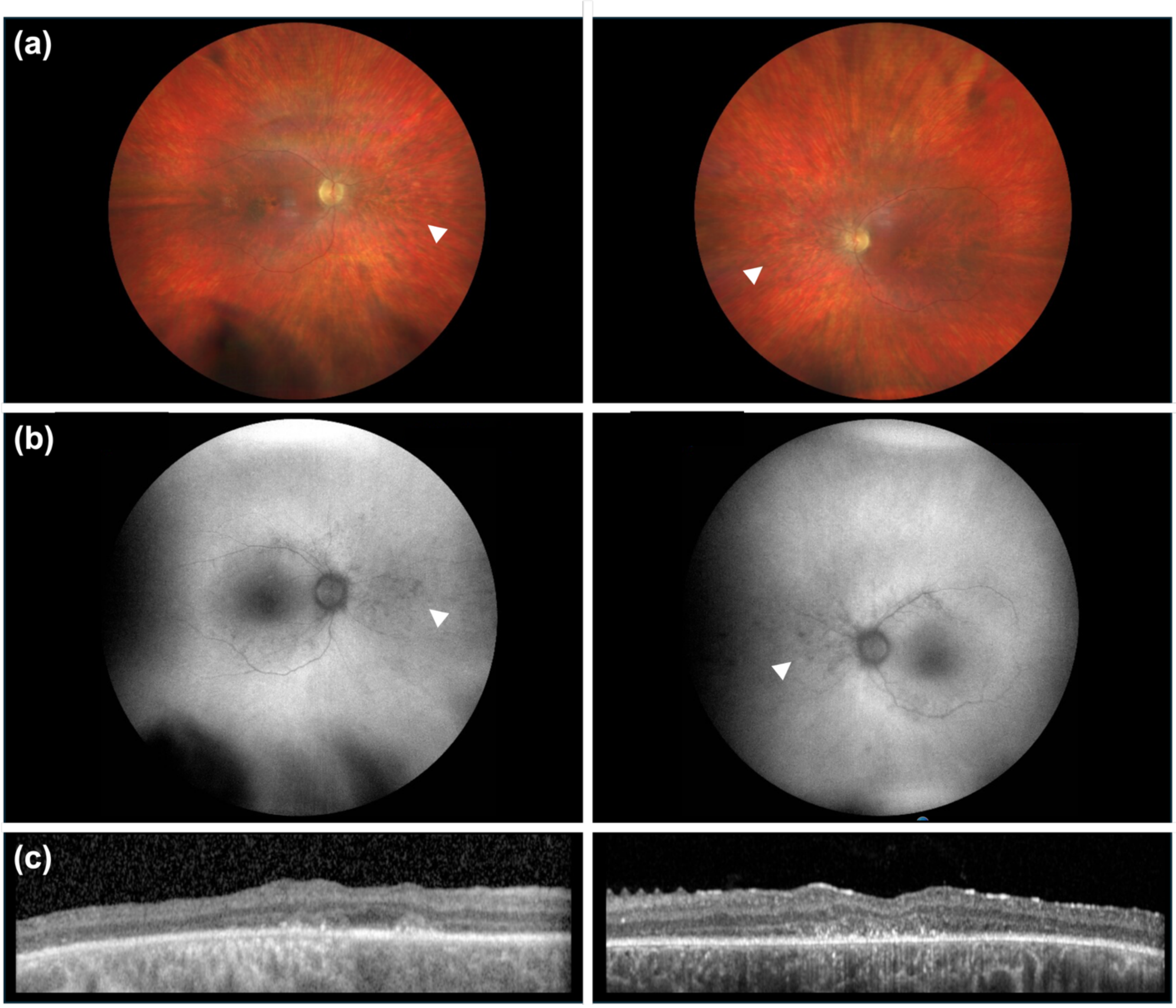

Supplementary Figure S3. Multimodal imaging of a Patient 3 - CRX-associated LCA due to a *p.Leu146CysfsTer41 (c.435delT)* likely pathogenic variant. 28-year-old male patient with a BCVA of 20/300 OD and 20/300 OS. HVF 30-2 showed diffuse depression OU. Genetic testing was performed with Molecular Vision Lab (Vision Panel v14). Fundus photography **(a)** and macula- and fundus autofluorescence of the posterior pole **(b)** shows widespread retinal atrophy with a distinct area of atrophy nasal to the disc (white arrowheads). **(c)** SD-OCT shows severe loss of the ellipsoid zone and outer segments.

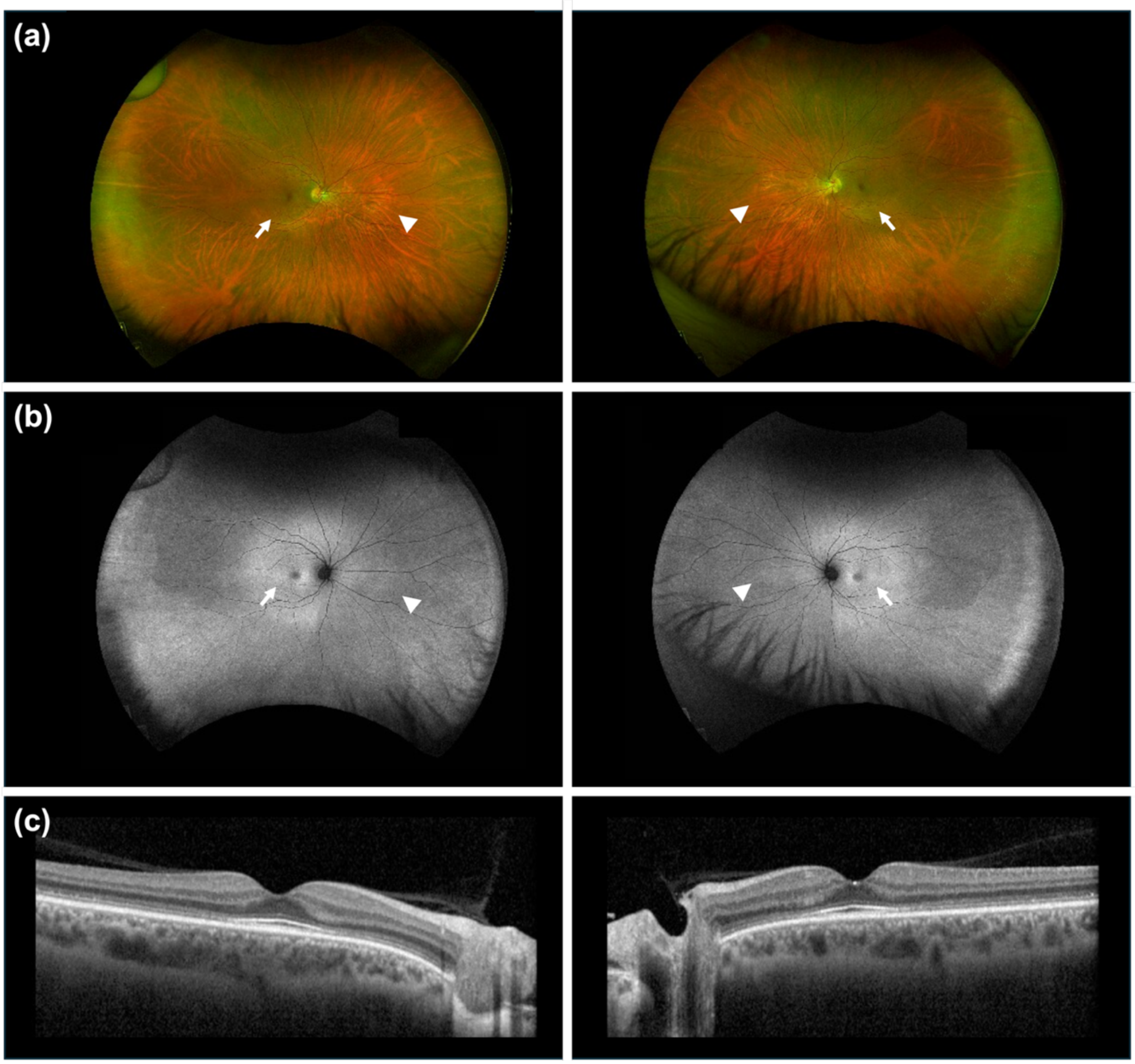

Supplementary Figure S4. Multimodal imaging of a Patient 5 - CRX-associated M/CRD due to a *c.100+3G>C* variant of uncertain significance. 39-year-old female patient with a BCVA of 20/20 OD and 20/20 OS. HVF 30-2 showed central scotoma and non-specific depression OU. Genetic testing was performed with Invitae (Inherited Retinal Dystrophy Panel). **(a)** Ultrawide-field fundus photography reveals an abnormal foveal light reflex (white arrows) and atrophic pigmentary changes nasal to the optic nerve (white arrowheads). **(b)** Fundus autofluorescence imaging shows hyper-autofluorescence in an early bull's eye pattern (white arrows) and very subtle areas of mixed autofluorescence nasal to the optic disc (white arrowheads) that corresponds to the nasal pigmentary changes in the color photographs. Nasal degeneration in this case is milder compared to other patients in the cohort. **(c)** SD-OCT shows subtle ellipsoid zone and outer segment irregularity. Of note, patient 5 is the daughter of patient 6.

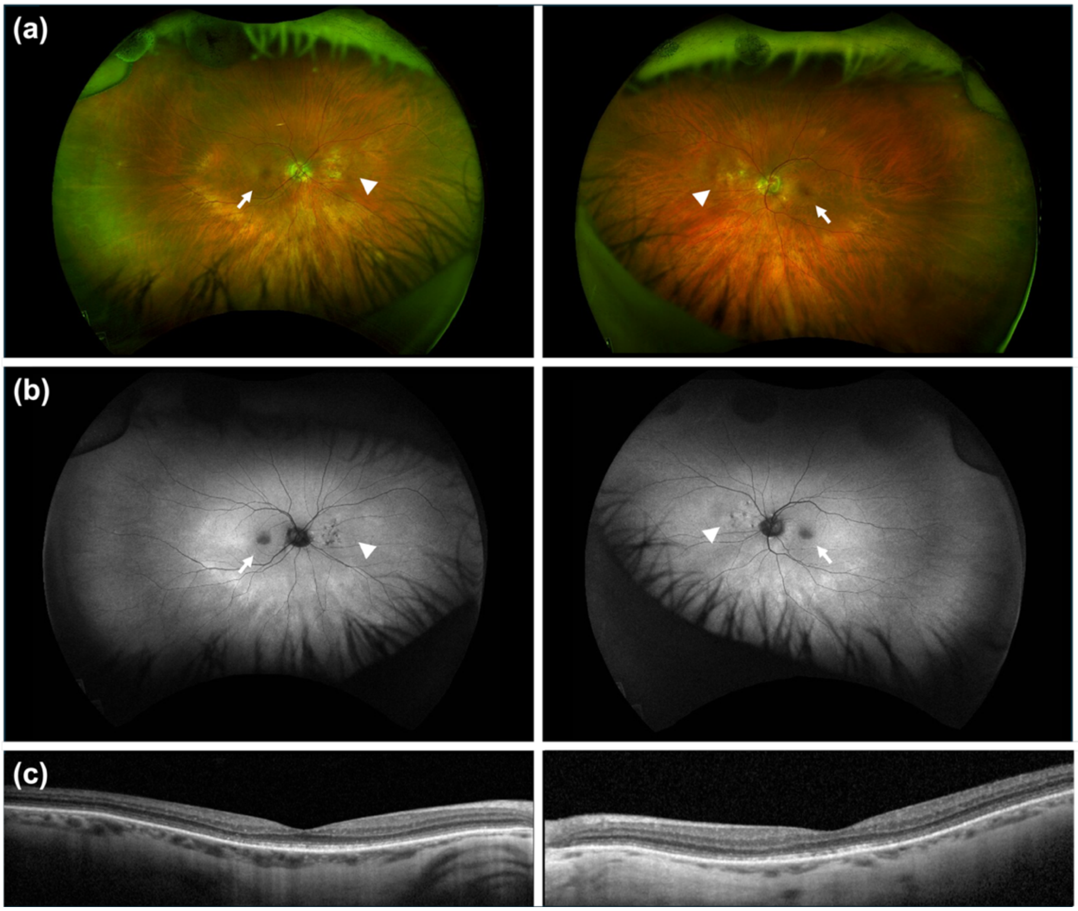

Supplementary Figure S5. Multimodal imaging of a Patient 6 - CRX-associated M/CRD due to a *c.100+3G>C* variant of uncertain significance. 60-year-old female patient with a BCVA of 20/25 OD and 20/40 OS. HVF 30-2 showed central scotoma OU and an area of decreased sensitivity corresponding to the peripapillary nasal retina, which was more pronounced OD than OS. Full-field ERG demonstrated normal rod responses as well as delayed and reduced cone responses. Multifocal ERG detected central depression, particularly in the perifoveal region. Genetic testing was performed with Invitae (Inherited Retinal Dystrophy Panel). Ultrawide-field fundus photography **(a)** demonstrates an abnormal foveal light reflex (white arrows) as well as a separate area of nasal retinal degeneration and pigmentary changes (white arrowheads). Fundus autofluorescence **(b)** reveals hypo-autofluorescence at the fovea (white arrows) and an area of mixed autofluorescent signal nasal to the disc (white arrowheads). There is also a ring of hyper-autofluorescence corresponding to pigmentary changes on the color photographs, most notably inferior and temporal to the macula. **(c)** SD-OCT captures subtle ellipsoid zone and outer segment irregularity. Of note, patient 6 is the mother of patient 5.

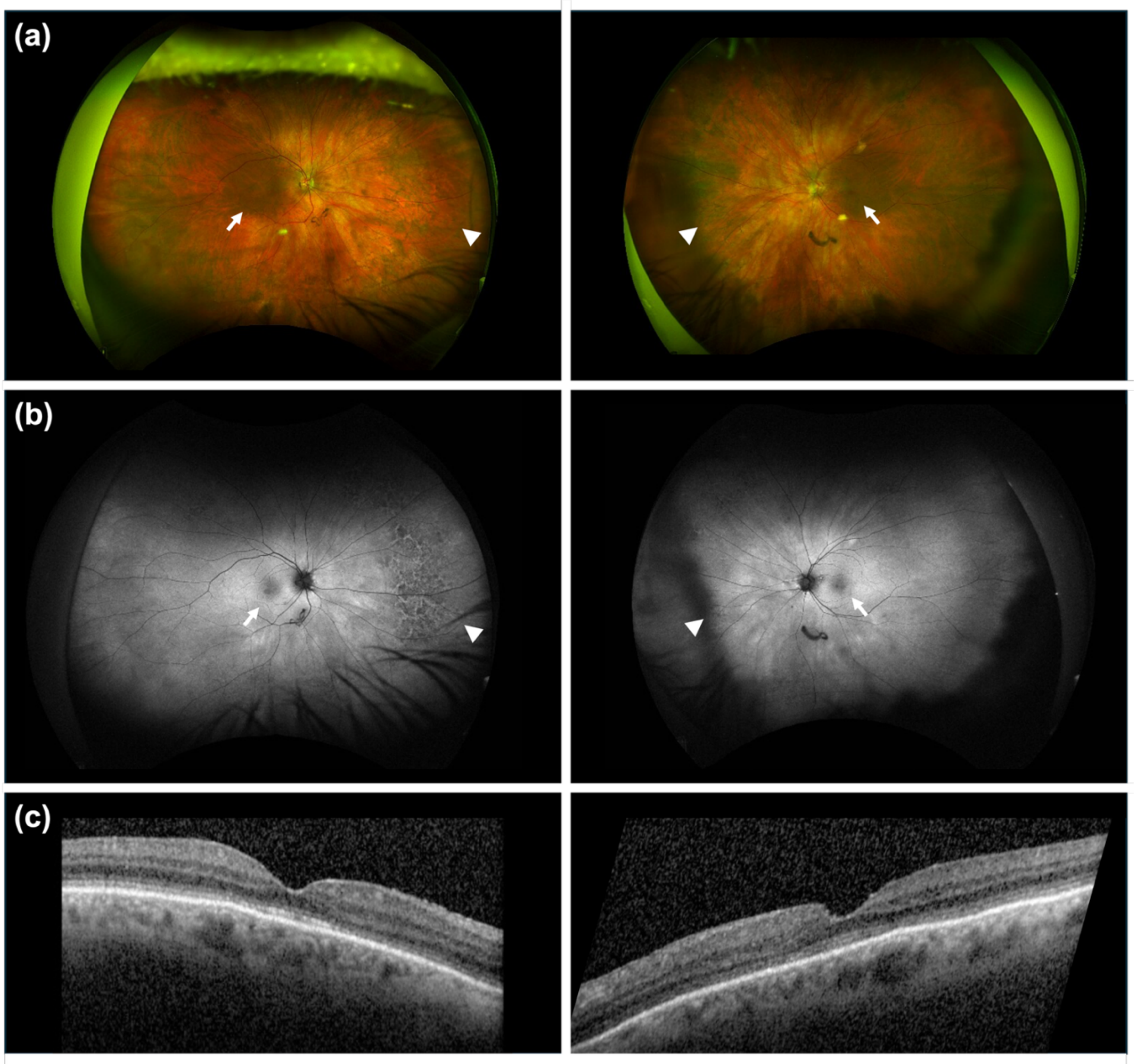

Supplementary Figure S6. Multimodal imaging of a Patient 7 - CRX-associated M/CRD due to a *c.100+3_100+5delGAGinsTTA* likely pathogenic variant. 68-year-old female patient with a BCVA of 20/30 OD and 20/25 OS. HVF 30-2 showed central scotoma OU. Full-field ERG demonstrated normal rod and cone responses. Genetic testing was performed with Molecular Vision Lab (Vision Panel v2). Ultrawide-field fundus photography **(a)** shows RPE irregularity in the macula and an abnormal foveal light reflex (white arrows) as well as a large area of pigmentary changes in the nasal retina (white arrowheads). Fundus autofluorescence **(b)** reveals abnormal hypo-autofluorescence around the fovea (white arrows) and an area of mixed autofluorescence signal in the nasal retina (white arrowheads). Nasal atrophy is more prominent in the right eye; however, there is an artifact in the images of the left eye that may be obscuring underlying retinal degeneration. **(c)** SD-OCT depicts mild outer segment and ellipsoid zone irregularity and an abnormal foveal contour in the left eye.

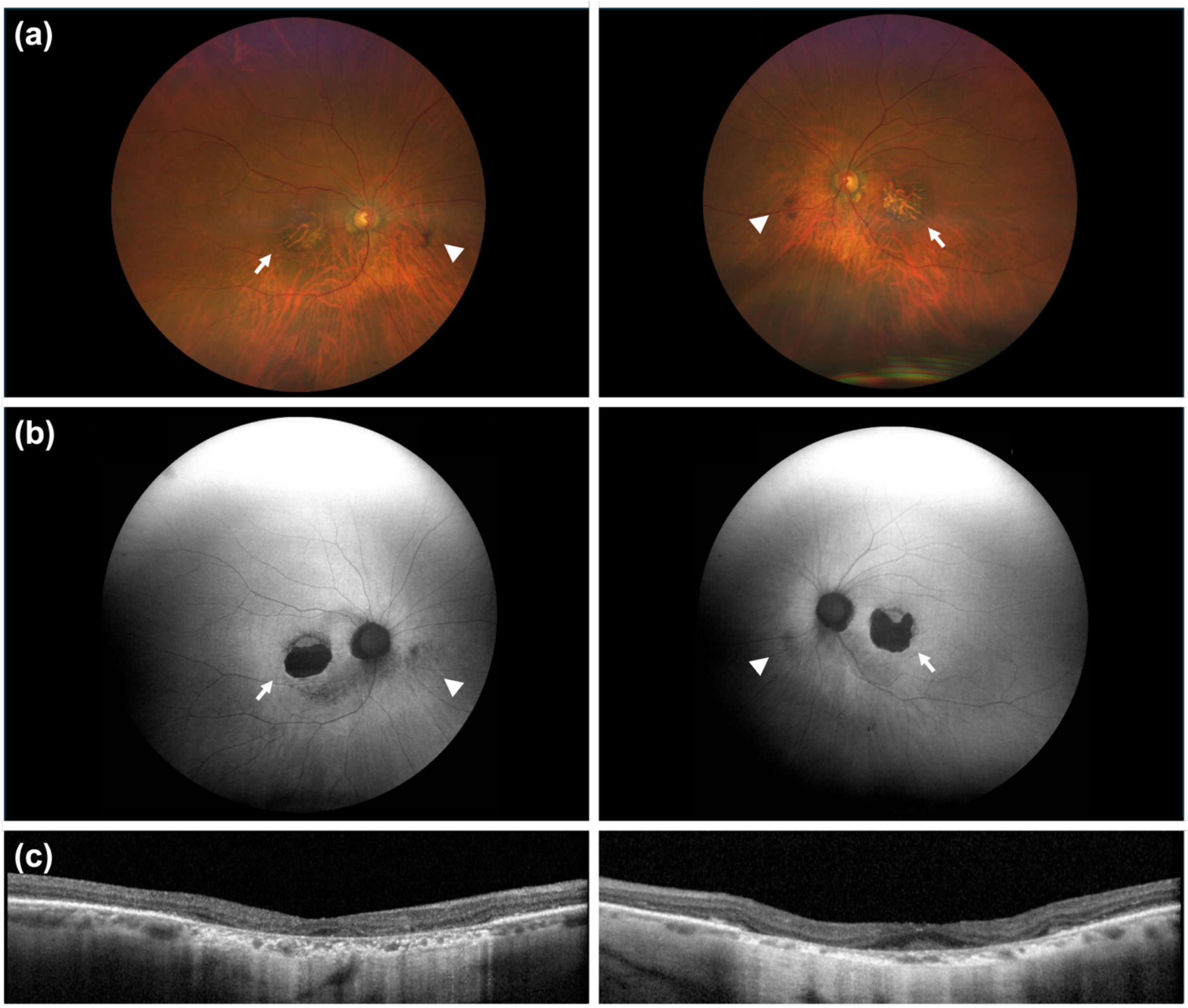

Supplementary Figure S7. Multimodal imaging of a Patient 8 - CRX-associated M/CRD due to a *p.Arg40Trp (c.118C>T)* pathogenic variant. 71-year-old female patient with a BCVA of 20/30 OD and 20/30 OS. HVF 30-2 showed central scotoma OU. Full-field ERG demonstrated decreased rod and cone responses. Genetic testing was performed with Blueprint Genetics (My Retina Tracker Program Panel). Ultrawide-field fundus photography **(a)** and fundus autofluorescence **(b)** demonstrate macular atrophy (white arrows) along with a distinct area of nasal retinal degeneration (white arrowheads). Additional atrophic changes are visible along the vascular arcades, particularly the inferior arcade. **(c)** SD-OCT reveals significant outer retinal thinning and RPE atrophy.

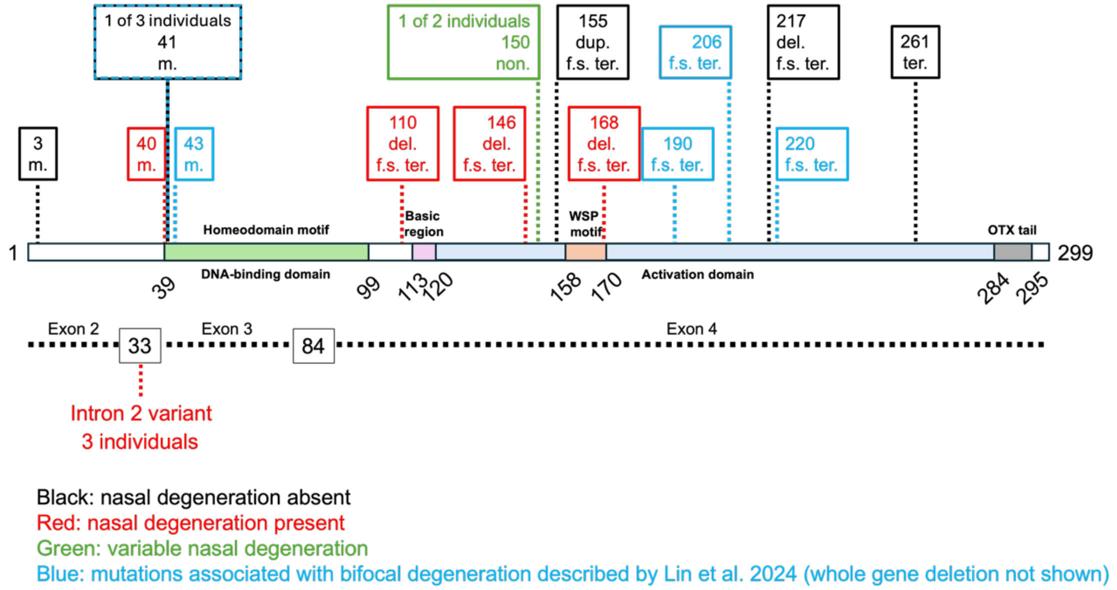

Supplementary Figure S8. Locations of Variants Associated with Nasal Degeneration Across the Protein and Exon Sequences, Including Variants Reported by Lin et al. The amino acid sequence is represented by the horizontal bar at the top, and the exon sequence is shown as a black dashed line across the bottom. Variants associated with the presence of nasal degeneration in our study are shown in red (n=8), and those reported by *Lin et al.* are shown in blue (n=6) (doi: 10.1038/s41433-024-03522-2) [10]. Variants associated with the absence of nasal degeneration in our study are shown in black (n=7). Variant p.Ser150Ter, shown in green, presented as M/CRD with nasal degeneration in one patient and as RCD/RP without nasal degeneration in a different patient in the cohort. Notably, the variant *p.Arg41Trp* was observed to be associated with nasal atrophy in the study by *Lin et al.*; however, this variant was not associated with nasal atrophy in our study. A list of variants can be found in Supplementary Table 1 (blue and black dashed box; third varid at this position was *p.Arg41Gln*). **Abbreviations:** m. = missense; non. = nonsense; del. = deletion; dup. = duplication; f.s. = frameshift; ter. = premature stop codon

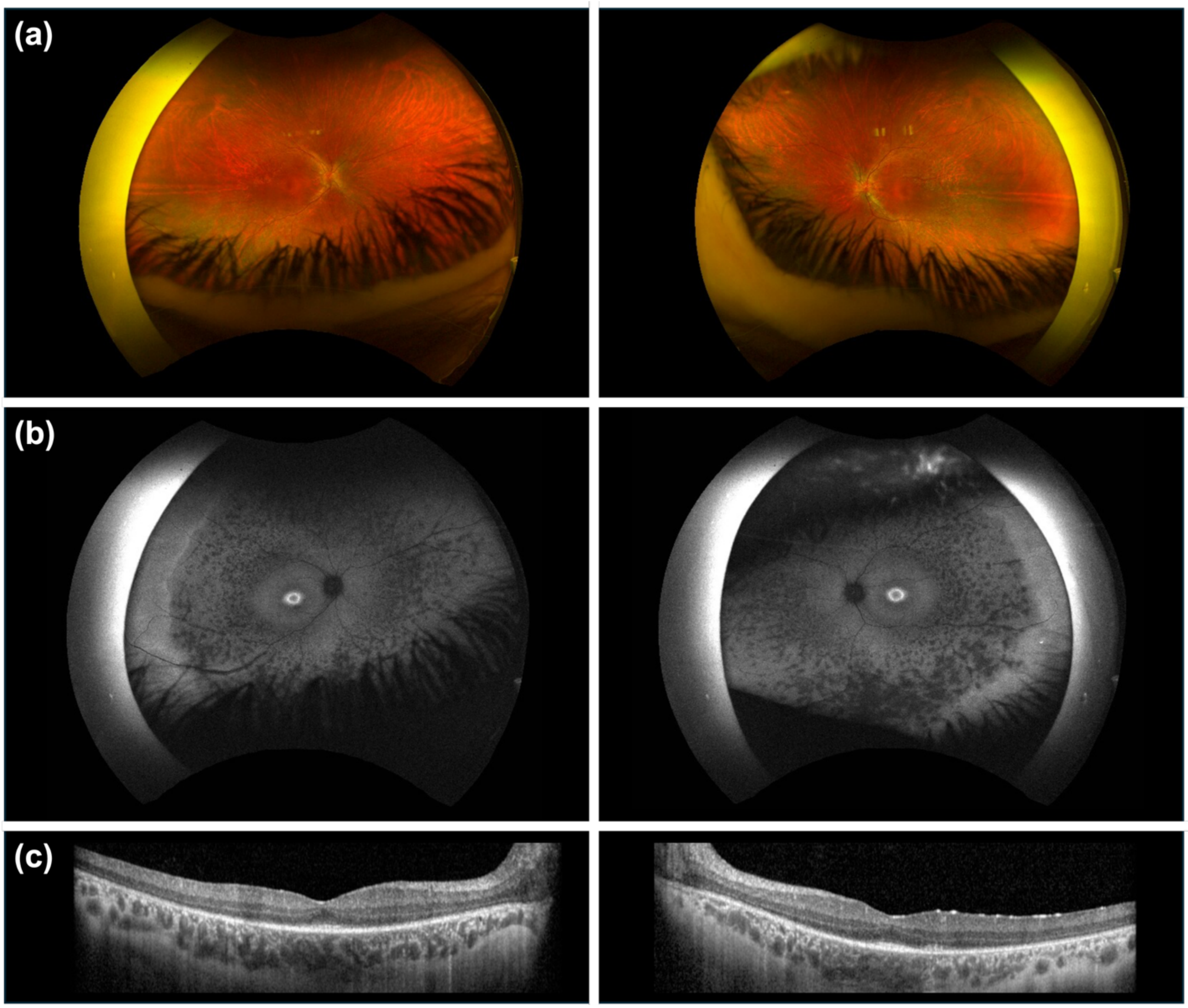

Supplementary Figure S9. Multimodal imaging of CRX-associated RCD/RP without focal nasal degeneration due to a *p.Ser150Ter (c.449C>G)* pathogenic variant. Ultrawide-field fundus photography **(a)** and fundus autofluorescence **(b)** show a bull's eye maculopathy with 360-degree peripheral retinal atrophy. An area of discrete nasal atrophy is not present. **(c)** SD-OCT shows ellipsoid zone and outer segment.

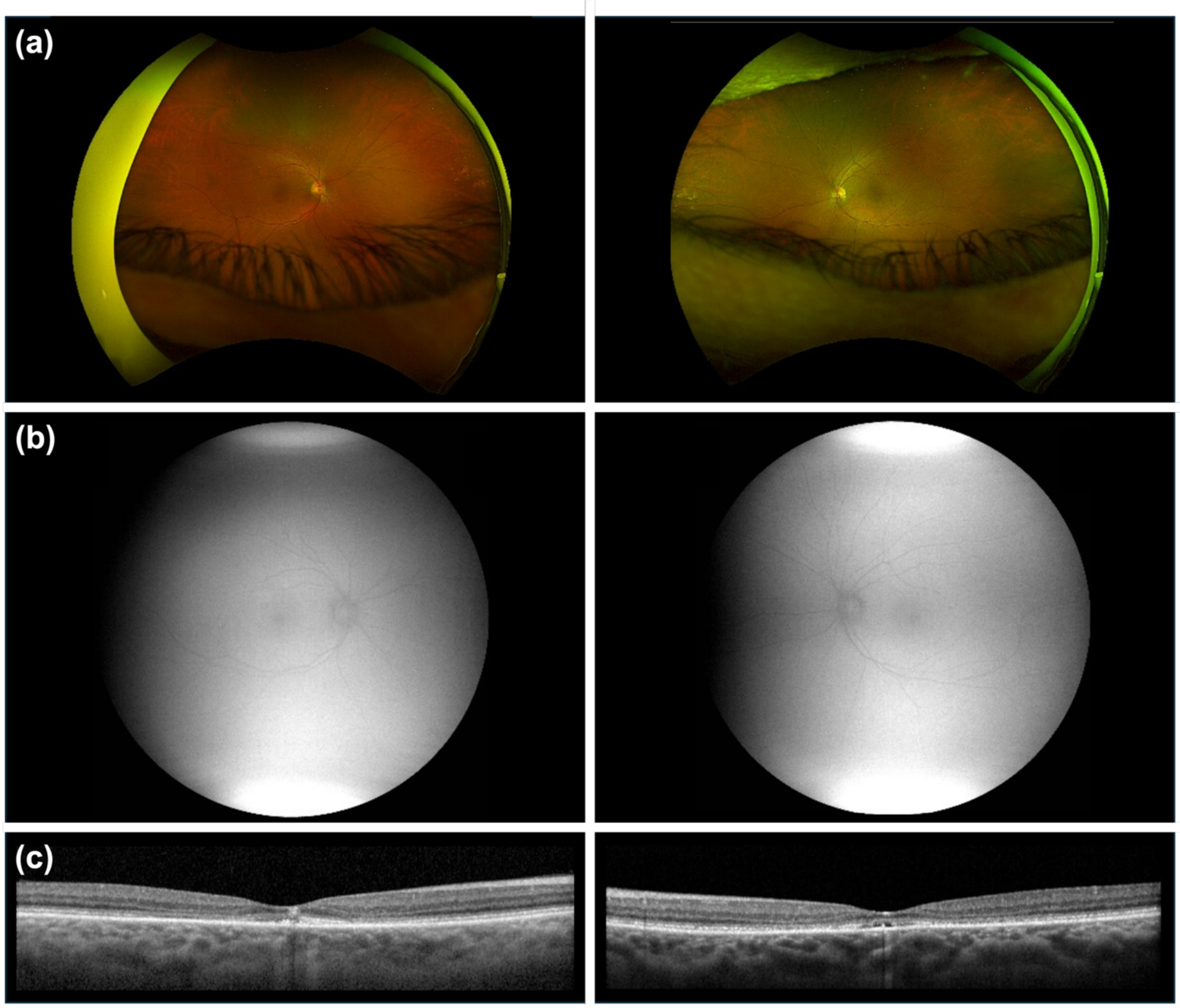

Supplementary Figure S10. Multimodal imaging of CRX-associated M/CRD without nasal degeneration due to a *p.Arg41Trp (c.121 C>T)* pathogenic variant. Ultrawide-field fundus photography **(a)** and fundus autofluorescence **(b)** show very subtle RPE irregularity and macular atrophy. Peripheral drusen can be seen far nasally; however, nasal degeneration is noticeably absent. **(c)** SD-OCT shows ellipsoid zone and outer segment loss in the foveal and parafoveal region.
